# Multimodal Integration of Brain Images for MRI-Based Diagnosis in Schizophrenia

**DOI:** 10.3389/fnins.2019.01203

**Published:** 2019-11-07

**Authors:** Raymond Salvador, Erick Canales-Rodríguez, Amalia Guerrero-Pedraza, Salvador Sarró, Diana Tordesillas-Gutiérrez, Teresa Maristany, Benedicto Crespo-Facorro, Peter McKenna, Edith Pomarol-Clotet

**Affiliations:** ^1^FIDMAG Hermanas Hospitalarias Research Foundation, Barcelona, Spain; ^2^Centro de Investigación Biomédica en Red de Salud Mental, Madrid, Spain; ^3^Hospital Benito Menni – Complex Assistencial en Salut Mental, Sant Boi de Llobregat, Spain; ^4^Hospital Universitario Marqués de Valdecilla, Universidad de Cantabria, Santander, Spain; ^5^Hospital Sant Joan de Déu, Barcelona, Spain

**Keywords:** multimodal integration, schizophrenia, machine learning, computer-aided diagnosis, convolutional neural network, lasso, ridge

## Abstract

Magnetic resonance imaging (MRI) has been proposed as a source of information for automatic prediction of individual diagnosis in schizophrenia. Optimal integration of data from different MRI modalities is an active area of research aimed at increasing diagnostic accuracy. Based on a sample of 96 patients with schizophrenia and a matched sample of 115 healthy controls that had undergone a single multimodal MRI session, we generated individual brain maps of gray matter vbm, 1back, and 2back levels of activation (*n*back fMRI), maps of amplitude of low-frequency fluctuations (resting-state fMRI), and maps of weighted global brain connectivity (resting-state fMRI). Four unimodal classifiers (Ridge, Lasso, Random Forests, and Gradient boosting) were applied to these maps to evaluate their classification accuracies. Based on the assignments made by the algorithms on test individuals, we quantified the amount of predictive information shared between maps (what we call redundancy analysis). Finally, we explored the added accuracy provided by a set of multimodal strategies that included post-classification integration based on probabilities, two-step sequential integration, and voxel-level multimodal integration through one-dimensional-convolutional neural networks (1D-CNNs). All four unimodal classifiers showed the highest test accuracies with the 2back maps (80% on average) achieving a maximum of 84% with the Lasso. Redundancy levels between brain maps were generally low (overall mean redundancy score of 0.14 in a 0–1 range), indicating that each brain map contained differential predictive information. The highest multimodal accuracy was delivered by the two-step Ridge classifier (87%) followed by the Ridge maximum and mean probability classifiers (both with 85% accuracy) and by the 1D-CNN, which achieved the same accuracy as the best unimodal classifier (84%). From these results, we conclude that from all MRI modalities evaluated task-based fMRI may be the best unimodal diagnostic option in schizophrenia. Low redundancy values point to ample potential for accuracy improvements through multimodal integration, with the two-step Ridge emerging as a suitable strategy.

## Introduction

In recent years, there has been growing interest in employing brain magnetic resonance imaging (MRI) datasets for medical diagnosis (Wang and Summers, 2012). Specifically, in the field of schizophrenia, a considerable number of studies have been carried out to evaluate the predictive power of machine learning algorithms based on MRI data (Wolfers et al., 2015; Arbabshirani et al., 2017). To improve the accuracy levels provided by unimodal data sources, some authors have explored ways to combine the information contained in images generated by different MRI modalities. These include methods with different levels of data integration and of a very different nature, ranging from simple post-classification majority-vote strategies to multimodal fusion techniques (Calhoun and Adali, 2009; Sui et al., 2013), including also multiple kernel learning (Peruzzo et al., 2015; Zu et al., 2016), multimodal Gaussian process classifiers (Young et al., 2013), and deep learning (Suk et al., 2014; Shi et al., 2018) among other techniques. Accordingly, multimodal MRI integration for clinical diagnosis is an open and dynamic area of research, but one that still requires investigation (Arbabshirani et al., 2017; Tulay et al., 2019).

Here, relying on two matched samples of patients with schizophrenia (*N* = 96) and healthy controls (*N* = 115) for which structural T1, task-based (*n*back task), and resting-state fMRI had been acquired in a single MRI session, we pursue three objectives: (i) to evaluate the differential discriminative power of brain maps derived from the different modalities; (ii) to quantify the degree to which the different types of images have similar or distinct predictive patterns; and (iii) to explore the added accuracy provided by a set of multimodal strategies based on different levels of data integration, including novel approaches such as a two-step data integration scheme and a one-dimensional-convolutional neural network (1D-CNN).

## Materials and Methods

### Sample, Image Acquisition, and Preprocessing

One hundred fifteen healthy controls and 96 patients with a diagnosis of schizophrenia according to DSM-IV criteria underwent a single MRI session where images were acquired with three different modalities. Both groups were matched for age (mean = 36.5, *SD* = 10.6, and range = 18–63 in controls; mean = 36.3, *SD* = 10.9, and range = 16–65 in patients), gender (67% of males in both groups), and premorbid IQ as estimated using the Word Accentuation Test (Test de Acentuación de Palabras, TAP) (Del Ser et al., 1997) (mean TAP controls 23.17, mean TAP patients 22.37, *t* = 1.2373, *p* = 0.2176). MRI acquisitions included a T1 structural image, a resting-state fMRI sequence, and an fMRI acquisition obtained during performance of the *n*back task (a working memory task). Acquisition parameters as well as a detailed description of the preprocessing steps applied to the images can be found in previous reports: T1 (Salvador et al., 2017b), resting-state fMRI (Salvador et al., 2017a), and the *n*-back task (Fuentes-Claramonte et al., 2019). Images from all modalities were coregistered to the same standard MNI152 T1 2 mm template. For the functional images, this involved an initial linear registration to the individual T1 image followed by a non-linear transformation to the standard MNI template. A generic mask only containing gray matter voxels was generated by applying a threshold on the gray matter probabilistic MNI template available from the FieldMap SPM toolbox^1^. Finally, only voxels that contained data in all modalities were included in the analyses. All participants gave written informed consent prior to participation. All the study procedures had been previously approved by the local research ethical committee and adhered to the Declaration of Helsinki.

### Brain Maps

From preprocessed images of the three different modalities, five different brain maps were generated. Individual gray matter voxel-based morphometry (GMVBM) maps were derived from the T1 images. GMVBM maps were selected from among other T1-related data formats because they had delivered optimal accuracy levels in a previous study (Salvador et al., 2017b).

**FIGURE 1:**
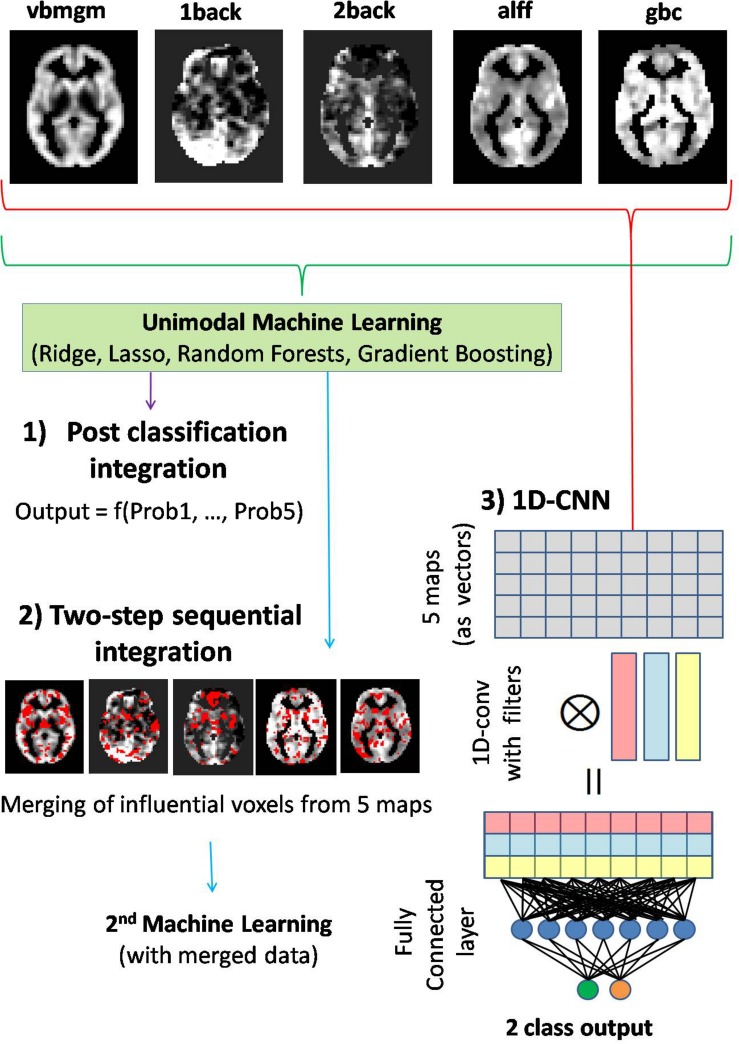
Diagram showing the three multimodal integrative strategies starting from the five different brain maps pictured on top of the figure. (1) Post-classification integration based on functions of output probabilities delivered by unimodal classifiers. (2) Two-step sequential integration based on an initial unimodal classification used to select the most informative voxels from each map, and followed by a second classification only considering the values of these voxels as inputs of the algorithm. (3) Voxel-level multimodal integration with 1D-convolutional neural networks (1D-CNNs). In this last approach, one-dimensional convolutions are applied across brain maps generating new maps that are combinations of the original ones. This is followed by a second fully connected layer linked to the two-node output layer (patient–control labels).

We used images containing the individual regression coefficients for the two main contrasts of the *n*-bask task: 1back-vs.-baseline and 2back-vs.-baseline (Gevins and Cutillo, 1993; Fuentes-Claramonte et al., 2019). Finally, from the resting-state fMRI sequences, we derived two brain maps containing complementary information. On the one hand, we calculated maps of the amplitude of low-frequency fluctuations (ALFF) (Zang et al., 2007), and on the other hand, we generated weighted global brain connectivity (GBC) maps, which are functional connectivity maps based on averaging correlations between each voxel and all the remaining gray matter voxels (Cole et al., 2010; Salvador et al., 2016). Once the five different maps had been generated (Figure 1, top), their resolution was downsampled to 4 mm to reduce computational and memory-storage costs (leading to each final map containing 19,660 voxels).

### Unimodal Machine Learning

Initially, we applied four different machine learning algorithms to evaluate the relative accuracy provided by the five brain maps. These algorithms included two linear additive classifiers: the Ridge and the Lasso logistic classifiers (Hastie et al., 2009). While the Ridge, with its L2 regularization, provides models in which all voxels had some weight, the Lasso (L1 regularization) provides sparse solutions with only few voxels contributing to the predictive model. Specifically, the functions contained in the glmnet package were applied through the R api (Friedman et al., 2010).

To lessen the linearity and additivity restrictions, two tree-based methods were further considered: a Random Forest and a Gradient Boosting algorithm (Hastie et al., 2009). For Random Forest, we considered 1000 trees and (number of voxels)^1/2^ variables per split, and for Gradient Boosting we carried out an internal cross-validation for the selection of optimal regularization and depth values. R libraries randomForest and gbm were used for the calculations.

A 10-fold cross-validation was used to obtain unbiased accuracy estimates of the different algorithms applied to the five brain maps by quantifying success rates in the test individuals. In each fold, prior to fitting the machine learning algorithms, linear models accounting for age and gender effects were fitted to the training sample. Later, coefficients of these models were applied to each corresponding test sample.

### Redundancy Assessment

A simple index to quantify the degree to which diagnostic predictive traits in a type of brain map are also present in another map (what we call here redundancy) can be easily derived from the success rates achieved in test samples. If Prob(M1) and Prob(M2) are the probabilities of successfully classifying a specific individual using brain maps of type 1 and type 2, we can simply quantify this redundancy with Prob(M2| M1) (i.e., the conditional probability of predicting successfully with map 2 provided that map 1 predicted the right class).

Conditional probabilities will be constrained by two limiting scenarios:

(1) Total redundancy: All predictive features of map 1 are contained in map 2. Then

(1) Prob⁢(M2|M1)=1

(i.e., if map 1 classifies correctly an individual, then map 2 will also do it).

(2) Complete independence: Maps do not share any predictive traits. Then

(2) Prob⁢(M2|M1)=Prob⁢(M2)

(i.e., the fact that map1 classifies correctly does not increase the probability of success with map2).

Since conditional probabilities will be in the [Prob(M2), 1] interval, a redundancy score (RSC) in the [0,1] interval will be given by

(3) RSC⁢(M2|M1)=(Prob⁢(M2|M1)-Prob⁢(M2))/(1-Prob⁢(M2))

Note that, obviously, RSC(M2| M1) will usually differ from RSC(M1| M2). RSCs will be useful for evaluating the potential increase in predictive power provided by merging both brain maps in a single classification (i.e., while low RSC values will indicate potential benefits from merging, RSC values close to 1 will not, as the maps will contain very similar predictive information). In our study, estimates of RSC for each pair of maps were obtained from the relative frequencies of individuals being correctly classified with each, or both, brain maps.

### Multimodal Integration

To take full advantage of the predictive power of the five brain maps, we followed three strategies with increasing levels of multimodal integration (Figure 1).

(1) Post-classification integration based on probabilities: After building unimodal classification algorithms based on the five brain maps and applying them to a test individual, this approach considered simple functions of the five outcome probabilities (delivered by the five brain maps) such as the mean probability or the maximum probability, finally classifying the individual according to this latter value. Alternatively, the log-odds of the same outcome probabilities but calculated for individuals in the training sets were considered as independent variables in a logistic model with diagnosis as the dependent variable. The fitted logistic model was then used to predict diagnosis for test individuals (using the log-odds of their outcome probabilities from unimodal algorithms as predictors).

(2) Two-step sequential integration: After applying unimodal machine learning on the training sets, the subset of “most relevant” voxels from each brain map was merged in a single dataset that, in turn, was used to train a final model. In Lasso and Ridge, relevance was defined by the magnitude of regression coefficients in the logistic model. Since Lasso is a sparse method, we selected all voxels with non-null coefficients, while for Ridge 20% of the voxels with the largest coefficients (in absolute value) were retained. For both tree-based methods, importance scores were used to select voxels. As Gradient Boosting showed a high level of sparsity, all voxels with non-zero importance scores were selected. For Random Forests the same 20% criterion as in Ridge was used.

(3) Voxel-level multimodal integration with 1D-CNNs: Unlike previous approaches, applying 1D convolutions at the voxel level across brain maps may permit finding optimal, within voxel combinations of modalities. In our study a two-layer neural network with one 1D convolutional layer including 10 filters and a standard fully connected layer with 50 hidden units was considered (Figure 1). Other parameters used were as follows: number of iterations = 50, batch size = 4, learning rate = 0.001, momentum = 0.9, wd = 0.0005, activation function = Relu. Network training was carried out with the mxnet library^2^.

The same 10-fold cross-validation scheme previously used for unimodal machine learning was applied in all analyses based on multimodal integration in order to secure unbiased accuracies from the test data.

## Results

### Unimodal Machine Learning

**FIGURE 2:**
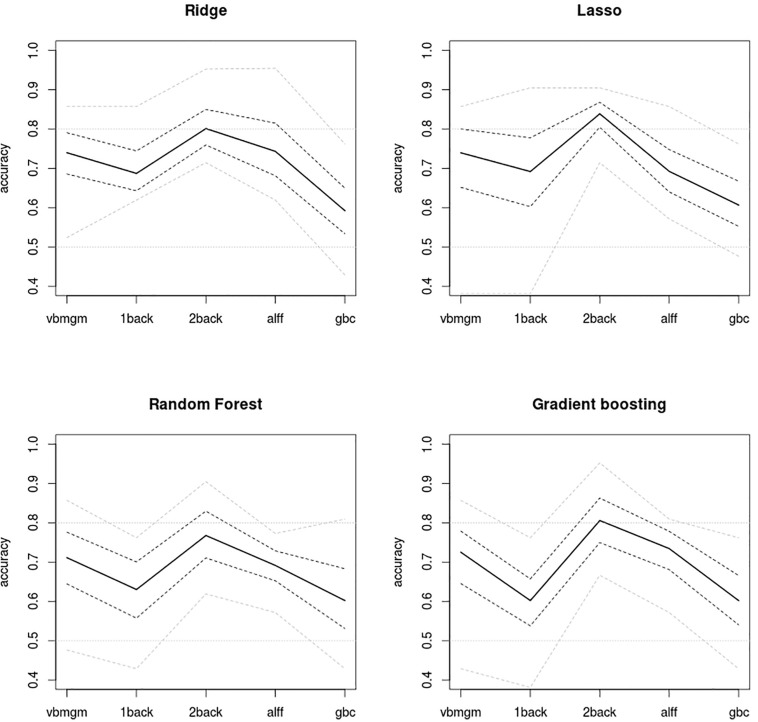
Plots of accuracy levels achieved by the four unimodal algorithms applied to the five brain maps. Mean accuracies extracted from test samples are shown by a continuous line. 2back maps show the strongest predictive power using all algorithms and GBC maps deliver the weakest. Gray dashed lines, 95% bootstrap intervals of mean accuracies and gray dotted lines, maximum and minimum accuracies delivered by the 10-fold scheme. 0.5 accuracy indicates chance accuracy (no real predictive power).

The main results from applying the different machine learning algorithms to the five brain maps are shown in Figure 2. In all four unimodal algorithms 2back maps showed the highest test accuracies (80% on average), achieving a maximum of 84% with Lasso. In contrast, GBC maps tended to deliver the lowest values (with an overall average of 60%). GMVBM (73%), 1back (65%), and ALFF (71%) maps had intermediate accuracy values. All accuracies are well >50%, indicating above-chance predictive power.

### Redundancy Assessment

**FIGURE 3:**
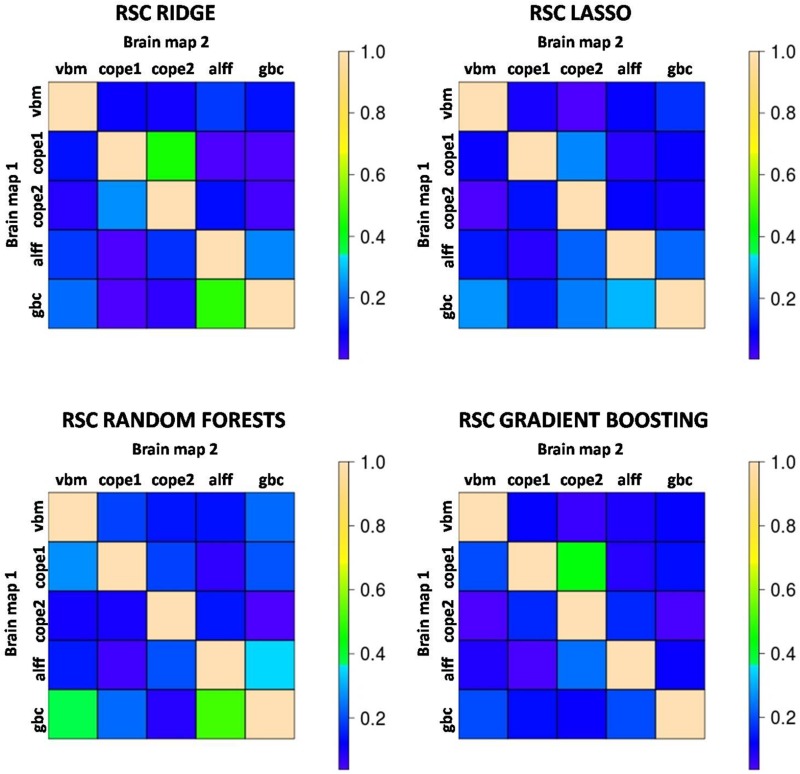
Color-coded values for the redundancy scores (RSC) that quantify the degree to which predictive features of Brain map 2 are also present in Brain map 1. An RSC value close to 1 indicates that map 2 brings almost no predictive information apart from that contained in map 1 (high redundancy) while a value of RSC close to 0 indicates that map 1 contains hardly any of the predictive patterns present in map 2 (both maps convey independent information). The highest RSCs tend to occur between maps derived from the same image modality, although these hardly ever reach values >0.50. Most of the RSCs are well below this number, indicating very low levels of redundancy and potential increases in accuracy through multimodal integration. cope1: 1back maps; cope2: 2back maps.

As shown in Figure 3, in general redundancy levels between brain maps were quite low, thus indicating that each brain map contained differential predictive information. As expected, the highest redundancies were observed between pairs of maps that came from the same MRI modality (i.e., 1back-2back maps and ALFF-GBC maps) but even these rarely reached RSCs of 0.5 [this only occurred for RSC(GBC|ALFF) = 0.51 with Random Forests]. In contrast, much lower redundancies were observed between maps derived from different MRI modalities, which in some cases showed almost complete independence in their predictive information, with RSC values as small as 0.002. Overall mean RSC was 0.14. In all, redundancy assessment indicated ample potential for accuracy improvements through multimodal integration. See the tables in the Supplementary Material 1 for numerical values of the RSC and the conditional probabilities.

### Multimodal Integration

**FIGURE 4:**
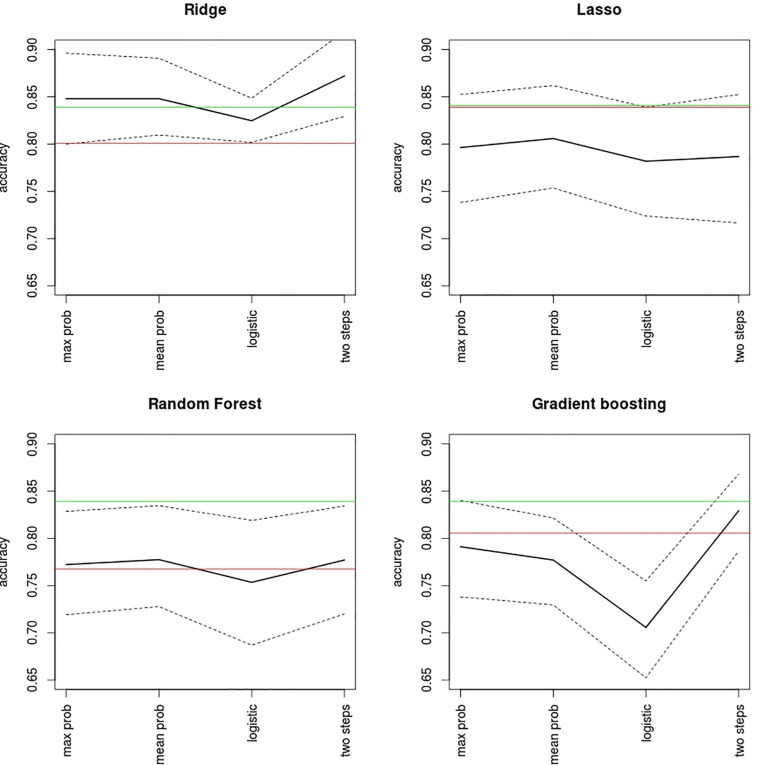
Accuracy levels reported by probability-based and two-step multimodal integration approaches. Mean accuracy (black line) and its bootstrap 95% confidence levels (dashed lines) are shown for each classifier. Mean accuracies achieved by the best unimodal classification (red line) and by the 1D-CNN algorithm (green line) are also shown for the purposes of comparison. Max prob, maximum output probability algorithm; mean prob, mean output probability algorithm; logistic, logistic model on the probabilities.

Accuracy levels achieved by the first two multimodal integration strategies (i.e., probability based and two-step sequential integration) are shown in Figure 4 together with the accuracies delivered by their respective best unimodal models and by the 1D-CNN classifier. Apart from the Ridge algorithm where the highest multimodal accuracies were achieved, all other multimodal accuracies were lower than that of the 1D-CNN classifier. Even so, the 1D-CNN classifier, with 84% accuracy, did not outperform the best unimodal result (i.e. Lasso on the 2back images, which also had 84% accuracy). The highest accuracies of all were obtained by the two-step Ridge classifier (87% accuracy) followed by the Ridge maximum and mean probability classifiers (both with 85% accuracy). From Figure 4 it can also be concluded that, while Ridge is an algorithm that seems to adjust well to multimodal integration, Lasso is not – as with the later multimodal strategies provide clearly lower accuracies than the best unimodal classifier. A similar pattern, although not as clear-cut as in Lasso was observed for Gradient Boosting, while Random Forests delivered multimodal accuracies similar to those from the best unimodal classifier.

**FIGURE 5:**
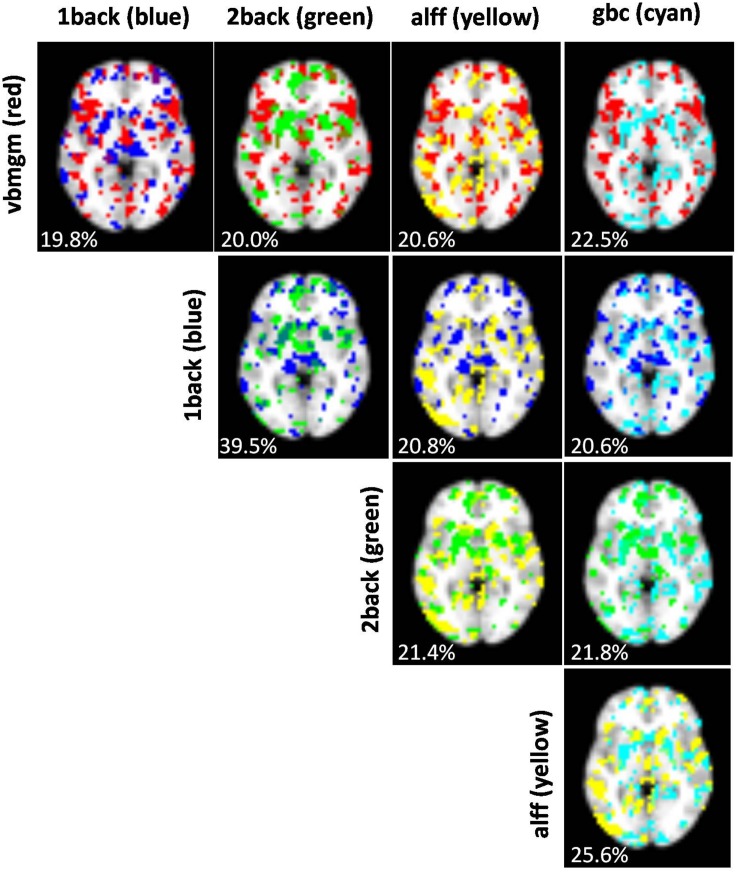
Images showing, for each pair of brain maps, the degree of overlap between voxels selected in the first step of the sequential Ridge algorithm. Percentages of overlap are given for each pair. Chance overlap under a scenario of complete spatial independence is 4%.

**FIGURE 6:**
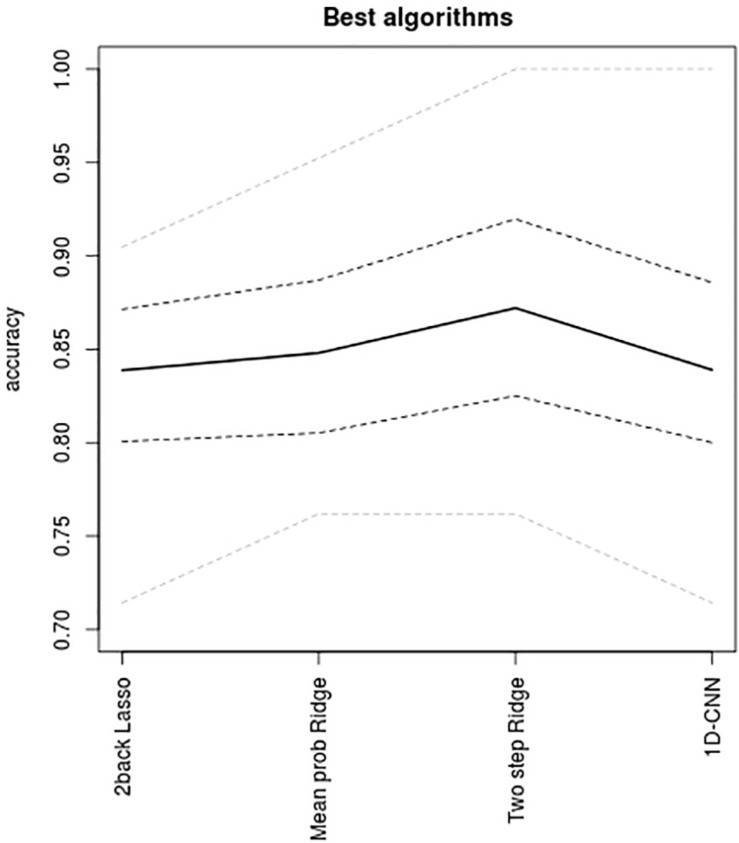
Accuracies achieved by the best-performing algorithm in the unimodal setting and for the three multimodal integrative strategies, namely, unimodal Lasso applied to the 2back maps, the mean of the output probabilities from Ridge (which performed equally well as the maximum probability algorithm), the two-step sequential Ridge algorithm, and the one-dimensional convolutional neural network. Continuous line, mean accuracies; gray dashed lines, 95% bootstrap intervals of mean accuracies; gray doted lines, maximum and minimum accuracies delivered by the 10-fold scheme.

The anatomical overlap between voxels selected in the first step of the sequential Ridge algorithm (the best performing algorithm) was remarkably low (Figure 5). As expected, the largest overlaps occurred between maps from the same image modalities (i.e., 1back-2back 39.5%, ALFF-GBC 25.6%) while the remaining pairs had values near 20%. Although this is clearly larger than the 4% overlap expected by complete chance (as the top 0.2 voxels were selected for each map), the observed levels of spatial overlap were, in general, low. This is in agreement with the low levels of redundancy reported in Figure 3. Supplementary Figure 1 shows the distribution of values for the regression coefficients selected in the first step of the two-step Ridge classifier. As a summary of all findings Figure 6 shows the highest test accuracy provided by the unimodal classifiers and by each one of the three multimodal integration strategies.

## Discussion and Conclusion

The main results from the four unimodal classifiers have given the highest predictive power to the 2back maps, suggesting that task-based fMRI may have a more relevant role than other MRI data sources in diagnostic prediction. On the other hand, although multimodal classifiers have not led to accuracies much higher than those provided by the unimodal classifiers, the low redundancy levels observed between modalities indicate that multimodal integration is a potentially valuable strategy that should be pursued in future studies.

It is remarkable that a single brain map (the 2back map) allowed for a high accuracy between patients and controls, and this is quite likely to be behind the best accuracies reached by the two-step Ridge classifier (87%). This figure is higher than the accuracies reported in recent multimodal classification studies in schizophrenia such as the 83% of Wu et al. (2018) and substantially higher than the 75% of Cabral et al. (2016). However, these studies did not include task-based fMRI data as in ours. It is noteworthy that classification studies involving patients with schizophrenia in which task fMRI data were used for prediction have usually reported high accuracy levels (Wolfers et al., 2015; Arbabshirani et al., 2017) although some of these studies, especially the older ones, may have been unreliable due to small sample sizes.

Interestingly, the best-performing multimodal classifiers in our study were based on the Ridge logistic regression, which is a rather simple and inflexible algorithm (it implies both additivity and linearity). This is in contrast with the general trend in the broad machine learning community, which favors more flexible methods based on neural networks and the gradient boosting algorithm (Chollet, 2018). Although success of the Ridge classifier could be explained by the specific nature of the information contained in the images, it is also probable that its performance over more flexible methods is partly due to the fact that the latter usually require substantially larger amounts of data to exceed other methods. In this sense, our proposed 1D-CNN could have been better trained, its structure could have been enriched with the inclusion of several hidden layers, and it might have delivered higher accuracies if a larger dataset was available.

Of interest are the low levels of redundancy reported between the different brain maps. These low redundancies, together with the small degree of anatomical overlap observed between voxels of the different maps as selected in the first step of the sequential Ridge algorithm, point to valuable exclusive information and to a wide margin of potential improvement by multimodal integration. In spite of this, such improvement was not large (from 84 to 87%), reflecting the intrinsic difficulties of efficiently combining information from different sources. At the same time, it may have been unrealistic to expect accuracies significantly larger than those achieved as the patient sample used in the study was rather heterogeneous, including individuals with a wide range of clinical profiles and of illness durations, ranging from subjects on a first episode to subjects with many years of illness evolution.

The profile of the patient sample has also other implications for the applicability of the developed algorithms. Ideally, the development of an MRI-based diagnostic tool should be based on and aimed at the appropriate target population, which in the case of schizophrenia would be individuals at risk or undergoing a first episode of psychosis. To date, however, there are few studies of multimodal MRI integration entirely based on samples of first-episode patients. Two examples are the study by Peruzzo et al. (2015), which used small sample sizes or the more recent study by Ebdrup et al. (2018), which found no predictive power in the MRI datasets.

Finally, it is also appropriate to consider another limitation of the study. As explained in the section “Materials and Methods,” downsampling to 4-mm voxels was made in order to reduce computational and memory-storage costs. Machine learning algorithms heavily rely on computational power, and their implementation may easily lead to the saturation of computational resources. In our study, this was especially relevant as, due to the multimodality, we were dealing with several datasets for each individual. Reduction of spatial resolution through voxel downsampling or ROI averaging may lead to some loss of relevant information. Still, it is difficult to evaluate the extent of this effect as downsampling differentially affects each modality and it has been shown to depend on the specific target groups (Gardumi et al., 2016; Mandelkow et al., 2017; Lancaster et al., 2018).

In summary, several conclusions may be drawn from our study. First, out of all MRI modalities evaluated, task-based fMRI appears to be the best option for unimodal diagnosis in schizophrenia. Secondly, the low levels of redundancy found between modalities suggest that multimodal integration is a potentially valuable strategy. Specifically, a simple and robust two-step algorithm based on the Ridge regression emerges as a suitable approach to multimodal diagnostic prediction in schizophrenia. Nevertheless, new studies based on samples of high-risk and first-episode patients will be required to develop valid multimodal MRI diagnostic tools in the disorder.

## Data Availability Statement

The raw data supporting the conclusions of this manuscript will be made available by the authors, without undue reservation, to any qualified researcher.

## Ethics Statement

The studies involving human participants were reviewed and approved by the Comité de Ética de la Investigación Clínica (CEIC-CEI) FIDMAG Hermanas Hospitalarias. The patients/participants provided their written informed consent to participate in this study.

## Author Contributions

RS, PM, EP-C, and BC-F: definition and design of the study. AG-P, SS, and EP-C: recruitment of participants. TM, AG-P, and EC-R: MRI data acquisition. RS, DT-G, and EC-R: data analysis and development of algorithms. RS, PM, EP-C, and BC-F: manuscript writing and revision.

## Conflict of Interest

The authors declare that the research was conducted in the absence of any commercial or financial relationships that could be construed as a potential conflict of interest.

## Footnotes

1 https://www.fil.ion.ucl.ac.uk/spm/toolbox/fieldmap

2 https://mxnet.apache.org/
