# Supplementary material for: Multimodal Integration of Brain Images for MRI-Based Diagnosis in Schizophrenia
Source: Front Neurosci. 2019 Nov 7;13:1203. doi: 10.3389/fnins.2019.01203 (PMC6855131; doi:10.3389/fnins.2019.01203)
Supplement: Supplementary file 1 [file Image_1.pdf]

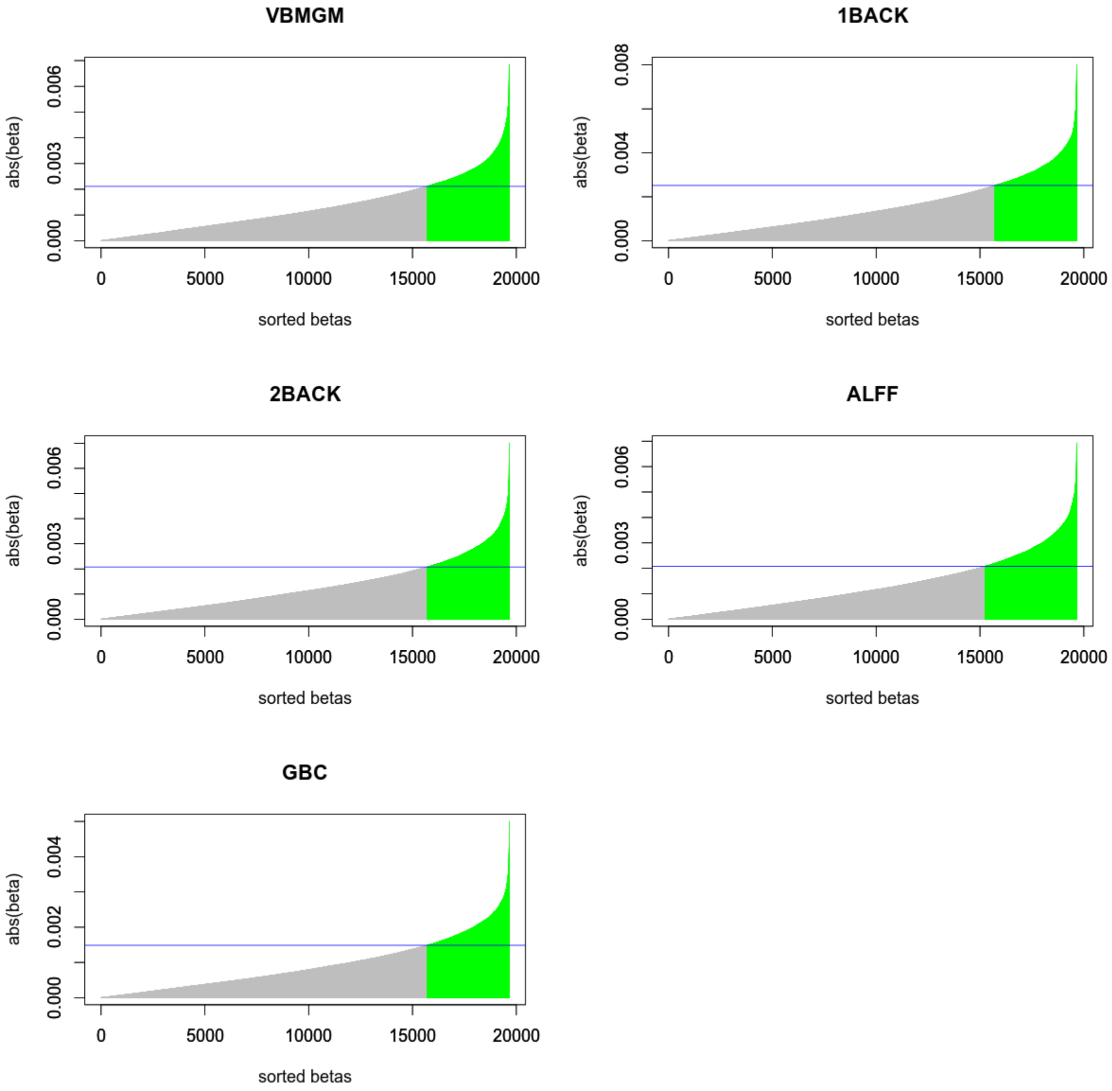

Supplementary Figure 1: Plots showing the absolute values for the regression coefficients (betas) assigned by the Ridge algorithm to each one of the 19,660 voxels contained in the five brain maps. The 20% of coefficients with largest values, which belonged to voxels selected for the second step of the sequential Ridge classifier, are shown in green. The blue line marks the inclusion threshold.
