# Supplementary material for: Multimodal Integration of Brain Images for MRI-Based Diagnosis in Schizophrenia
Source: Front Neurosci. 2019 Nov 7;13:1203. doi: 10.3389/fnins.2019.01203 (PMC6855131; doi:10.3389/fnins.2019.01203)
Supplement: Supplementary file 2 [file Data_Sheet_1.docx]

**Table 1**: Levels of redundancy between pairs of brain maps in terms of the amount of predictive information shared between them when Ridge logistic models were applied unimodally. PROB: estimated probability of correct classification given by Brain map 2, CON: estimated conditional probability of correct classification by Brain map 2 when Brain map 1 has correctly classified, RSC: Redundancy score as defined in Eq. 3 of main text.

| RIDGE | | | | | | |
| --- | --- | --- | --- | --- | --- | --- |
| Brain map 2 (M2) | | | | | | |
| Brain map 1 (M1) |  | GMVBM | 1BACK | 2BACK | ALFF | GBC |
|  | GMVBM |  | PROB: 0.687  CON: 0.712  RSC: 0.078 | PROB: 0.801  CON: 0.814  RSC: 0.066 | PROB: 0.744  CON: 0.782  RSC: 0.148 | PROB: 0.592  CON: 0.635  RSC: 0.104 |
|  | 1BACK | PROB: 0.739  CON: 0.766  RSC: 0.1 |  | PROB: 0.801  CON: 0.89  RSC: 0.446 | PROB: 0.744  CON: 0.745  RSC: 0.003 | PROB: 0.592  CON: 0.593  RSC: 0.002 |
|  | 2BACK | PROB: 0.739  CON: 0.751  RSC: 0.047 | PROB: 0.687  CON: 0.763  RSC: 0.243 |  | PROB: 0.744  CON: 0.769  RSC: 0.098 | PROB: 0.592  CON: 0.598  RSC: 0.013 |
|  | ALFF | PROB: 0.739  CON: 0.777  RSC: 0.145 | PROB: 0.687  CON: 0.688  RSC: 0.002 | PROB: 0.801  CON: 0.828  RSC: 0.136 |  | PROB: 0.592  CON: 0.688  RSC: 0.234 |
|  | GBC | PROB: 0.739  CON: 0.792  RSC: 0.202 | PROB: 0.687  CON: 0.688  RSC: 0.003 | PROB: 0.801  CON: 0.808  RSC: 0.035 | PROB: 0.744  CON: 0.864  RSC: 0.469 |  |

**Table 2**: As Table 1 but with results delivered by the Lasso logistic classifier.

| LASSO | | | | | | |
| --- | --- | --- | --- | --- | --- | --- |
| Brain map 2 (M2) | | | | | | |
| Brain map 1 (M1) |  | GMVBM | 1BACK | 2BACK | ALFF | GBC |
|  | GMVBM |  | PROB: 0.692  CON: 0.712  RSC: 0.064 | PROB: 0.839  CON: 0.84  RSC: 0.005 | PROB: 0.692  CON: 0.718  RSC: 0.084 | PROB: 0.607  CON: 0.66  RSC: 0.136 |
|  | 1BACK | PROB: 0.739  CON: 0.76  RSC: 0.08 |  | PROB: 0.839  CON: 0.877  RSC: 0.235 | PROB: 0.692  CON: 0.705  RSC: 0.044 | PROB: 0.607  CON: 0.637  RSC: 0.077 |
|  | 2BACK | PROB: 0.739  CON: 0.74  RSC: 0.003 | PROB: 0.692  CON: 0.723  RSC: 0.101 |  | PROB: 0.692  CON: 0.718  RSC: 0.083 | PROB: 0.607  CON: 0.633  RSC: 0.066 |
|  | ALFF | PROB: 0.739  CON: 0.767  RSC: 0.107 | PROB: 0.692  CON: 0.705  RSC: 0.044 | PROB: 0.839  CON: 0.87  RSC: 0.192 |  | PROB: 0.607  CON: 0.685  RSC: 0.199 |
|  | GBC | PROB: 0.739  CON: 0.805  RSC: 0.251 | PROB: 0.692  CON: 0.727  RSC: 0.112 | PROB: 0.839  CON: 0.875  RSC: 0.224 | PROB: 0.692  CON: 0.781  RSC: 0.29 |  |

**Table 3**: As Table 1 but with results delivered by the Rando Forest algorithm.

| RANDOM FORESTS | | | | | | |
| --- | --- | --- | --- | --- | --- | --- |
| Brain map 2 (M2) | | | | | | |
| Brain map 1 (M1) |  | GMVBM | 1BACK | 2BACK | ALFF | GBC |
|  | GMVBM |  | PROB: 0.63  CON: 0.7  RSC: 0.188 | PROB: 0.768  CON: 0.8  RSC: 0.139 | PROB: 0.692  CON: 0.733  RSC: 0.134 | PROB: 0.602  CON: 0.693  RSC: 0.23 |
|  | 1BACK | PROB: 0.711  CON: 0.789  RSC: 0.272 |  | PROB: 0.768  CON: 0.812  RSC: 0.191 | PROB: 0.692  CON: 0.714  RSC: 0.073 | PROB: 0.602  CON: 0.684  RSC: 0.207 |
|  | 2BACK | PROB: 0.711  CON: 0.741  RSC: 0.103 | PROB: 0.63  CON: 0.667  RSC: 0.098 |  | PROB: 0.692  CON: 0.735  RSC: 0.138 | PROB: 0.602  CON: 0.617  RSC: 0.039 |
|  | ALFF | PROB: 0.711  CON: 0.753  RSC: 0.147 | PROB: 0.63  CON: 0.651  RSC: 0.055 | PROB: 0.768  CON: 0.815  RSC: 0.204 |  | PROB: 0.602  CON: 0.74  RSC: 0.346 |
|  | GBC | PROB: 0.711  CON: 0.819  RSC: 0.374 | PROB: 0.63  CON: 0.717  RSC: 0.233 | PROB: 0.768  CON: 0.787  RSC: 0.085 | PROB: 0.692  CON: 0.85  RSC: 0.514 |  |

**Table 4**: As Table 1 but with results generated by the Gradient Boosting algorithm.

| GRADIENT BOOSTING | | | | | | |
| --- | --- | --- | --- | --- | --- | --- |
| Brain map 2 (M2) | | | | | | |
| Brain map 1 (M1) |  | GMVBM | 1BACK | 2BACK | ALFF | GBC |
|  | GMVBM |  | PROB: 0.602  CON: 0.647  RSC: 0.113 | PROB: 0.806  CON: 0.817  RSC: 0.058 | PROB: 0.735  CON: 0.758  RSC: 0.089 | PROB: 0.602  CON: 0.647  RSC: 0.113 |
|  | 1BACK | PROB: 0.725  CON: 0.78  RSC: 0.198 |  | PROB: 0.806  CON: 0.89  RSC: 0.433 | PROB: 0.735  CON: 0.756  RSC: 0.08 | PROB: 0.602  CON: 0.654  RSC: 0.13 |
|  | 2BACK | PROB: 0.725  CON: 0.735  RSC: 0.037 | PROB: 0.602  CON: 0.665  RSC: 0.158 |  | PROB: 0.735  CON: 0.776  RSC: 0.158 | PROB: 0.602  CON: 0.618  RSC: 0.04 |
|  | ALFF | PROB: 0.725  CON: 0.748  RSC: 0.085 | PROB: 0.602  CON: 0.619  RSC: 0.044 | PROB: 0.806  CON: 0.852  RSC: 0.236 |  | PROB: 0.602  CON: 0.645  RSC: 0.109 |
|  | GBC | PROB: 0.725  CON: 0.78  RSC: 0.198 | PROB: 0.602  CON: 0.654  RSC: 0.13 | PROB: 0.806  CON: 0.827  RSC: 0.109 | PROB: 0.735  CON: 0.787  RSC: 0.199 |  |
